# Alteration of functional connectivity and network properties after stereo-electroencephalography guided radiofrequency thermocoagulation

**DOI:** 10.1186/s41016-026-00428-8

**Published:** 2026-03-12

**Authors:** Danyi Shen, Lanling Zhou, Nianshun Liao, Sixun Yu, Xin Chen, Haifeng Shu

**Affiliations:** 1 Department of Neurosurgery, College of Medicine, The General Hospital of Western Theater Command, Southwest Jiaotong University, Chengdu, 610031 China; 2 Department of Neurosurgery, General Hospital of Western Theater Command of PLA, Chengdu, Sichuan 610083 China; 3 Department of Neurosurgery, The General Hospital of Western Theater Command, Chengdu Medical College, Chengdu, 610500 China

**Keywords:** Functional connectivity, Graph theory, Stereo-electroencephalography, Radiofrequency thermocoagulation, Epileptogenic zone

## Abstract

**Background:**

Stereo-electroencephalography guided radiofrequency thermocoagulation (RF-TC) aims at changing epileptogenic networks to achieve therapeutic purpose. However, the functional connectivity mechanism of RF-TC remains unknown. We sought to determine the effects of RF-TC on functional connectivity and the relationship between these variations and the clinical outcome.

**Methods:**

For this retrospective cohort study, we analyzed resting-state stereoelectroencephalography (SEEG) data segments to assess functional connectivity across sampling areas in seventeen epilepsy patients. We analyzed the variance of functional connectivity and graph theory indicators and assessed the relationship between variation and clinical response to RF-TC.

**Results:**

We found decreased functional connectivity both within and between epileptogenic zone in alpha band (*p* < 0.05) after RF-TC. We also discovered the alteration of most graph theory properties in the alpha band. Moreover, within connectivity and betweenness were significantly decreased in alpha band in the non-improvement group (*p* < 0.05), while clustering coefficient showed opposite change in the improvement group (*p* < 0.05). Eventually, compared to improvement group, we discovered a greater decrease of within connectivity of alpha band in the epileptogenic zone (*p* < 0.01).

**Conclusion:**

The research on network changes after radiofrequency thermocoagulation (RF-TC) is still an evolving field. Our research results indicate that significant changes occurred in functional connectivity and network characteristics in specific frequency bands and brain regions after RF-TC. Notably, the reduction in the internal connectivity within the alpha frequency band of the epileptic lesion not only provides early electrophysiological feedback for RF-TC, but also serves as a potential indicator for evaluating clinical response and prognosis.

**Supplementary Information:**

The online version contains supplementary material available at 10.1186/s41016-026-00428-8.

## Background

The goal of stereoelectroencephalography (SEEG)-guided radiofrequency thermocoagulation (RF-TC) in patients with focal drug-resistant epilepsy is to modify local brain networks [1, 2]. It is conducted after SEEG recording has completed, creating thermocoagulative lesions with the same SEEG electrodes in the target areas. As a result, SEEG appears to be more than only a diagnostic treatment, but also a potential therapeutic alternative in epilepsy patients [3, 4].

According to previous studies, the clinical outcomes of RF-TC were either inferior to or comparable to those of conventional resection operations [2, 5]. It has previously been observed that the reduction of the rate of interictal spikes and highfrequency oscillation may be related to clinical improvement after the RF-TC procedure [6]. Another concept was that RF-TC enhanced clinical efficacy by suppressing the original epileptogenic network [7]. Existing research has identified the critical role played by functional connectivity alteration in the effectiveness of classic epilepsy resection procedures. One study discovered a correlation between surgical outcomes and the functional connectivity rating [8]. Moreover, individuals with more nodes showing high betweenness centrality in pre-surgical postictal and interictal networks have worse seizure outcomes after resection, even though excision of nodes with a high degree of connectivity in some networks was related to recurrent seizures [9]. Besides, eigenvector centrality has been recognized as a biomarker of isolated electrodes in electrocorticographic recordings that could determine the seizure zone [10]. While research on SEEG-guided RF-TC was traditionally focused on local electrophysiological effects, a growing body of recent studies has begun to elucidate its broader impact on functional connectivity and graph theory metrics. Recent evidence suggests that RF-TC acts as a network-level intervention rather than a purely focal lesioning tool, with significant post-procedural changes observed in both local and distant brain networks.

Based on prior research, the purpose of this study was to quantify the effects of RF-TC on functional connectivity and to assess the relationship between these changes and t clinical outcomes following RF_TC procedure.

## Methods

### Patients

In this study, we initially screened a total of 25 consecutive patients with medically refractory epilepsy from the database of the Western Theater Command General Hospital in the period July 2019 to November 2021. Patients meeting the following criteria were included in this study: (1) underwent SEEG and video-EEG monitoring at our institution; (2) recorded at least 15 min SEEG data before and after RF-TC; (3) completed at least 6 months of follow-up after RF-TC. Among them, 8 patients were excluded from the final analysis due to the following reasons: (1) sufficient SEEG recording duration or poor signal quality post-RF-TC (*n* = 5); and (2) loss to follow-up or follow-up duration of less than 6 months (*n* = 3). Consequently,, only 17 patients were included in in the final cohort. All procedures were approved by the Ethics Committee of Western Theater Command General Hospital and written informed consent was obtained for SEEG electrode implantation and the subsequent RF-TC procedure.

Regarding the therapeutic benefits of RF-TC, patients were considered to have a favorable clinical response if they achieved at least a 50% reduction in seizure frequency or seizure freedom, consistent with like the description in previous studies [6]. In detail, the improvements should persist for at least 1 month in patients with daily seizures before RF-TC, 2 months for patients with weekly seizures, and 6 months in those with monthly seizures. For patients who eventually required subsequent resective surgery due to insufficient seizure control after RF-TC, the final follow-up day was defined as the day immediately preceding that surgery to ensure that only the effects of the thermocoagulation were evaluated. During the follow-up, we confirmed the follow-up information with patients and their families respectively to prevent possible recall bias.

### SEEG and thermocoagulation procedures

Patients underwent SEEG recording according to information available from non-invasive examinations (such as high-density scalp video-EEG monitoring, high-resolution MRI, neuropsychological testing, positron emission tomography) and clinical hypotheses about the location of the epileptogenic zone [11]. Intracerebral multiple contact electrodes (Beijing HKHS Healthcare co., ltd, China, 8–16 contact, length: 2 mm, diameter: 0.8 mm, 1.5 mm apart) were implanted for SEEG exploration. A postoperative computed tomography (CT) scan was used to confirm that there were no complications (such as bleeding) and spatial localization of implantation. We used Brainstorm, an open-source Matlab (version 2018b; MathWorks Inc, Natick, Massachusetts) toolbox, to coregister the CT and MRI to precisely localize the contact along the electrode's trajectory [12]. The Desikan-Killiany Atlas was used to determine the anatomical position of all contacts. Electrode contacts outside the brain, in white matter, or in cerebrospinal fluid were exclued.

The video-SEEG recordings were continued for as long as necessary to capture multiple habitual seizures. SEEG signals were acquired using a Natus video-EEG system (Natus Medical Incorporated) at a sampling rate of 2048 Hz (one patient was recorded at 512 Hz). RF-TC was performed at the end of the SEEG recordings. Contact used for RF-TC were selected based on their involvement in seizure onset or early propagation zone as described previously [13]. However, selected contacts were eliminated from the RF-TC if they were in a functionally cortex or close to vascular structures. Each RF-TC lesion was produced a pair of contiguous contacts connected to radiofrequency lesion generator equipment (R-2000B, Beiqi, Beijing, China). All RF-TC procedures were performed under video-SEEG monitoring. If the impedance of the contacts utilized for RF-TC did not considerably reduce following RF-TC, then subsequent RF-TC treatments were performed (as shown in Supplementary Fig. S1).

### SEEG signal analysis

15 min of inter-ictal SEEG data in the awake, resting state were collected before the beginning of the RF-TC session and 15 min immediately after the last RF-TC procedure. Raw SEEG data were then preprocessed in Brainstorm (bandpass filter, 0.16–80 Hz; notch filter, 50 Hz). Frequencies above 500 Hz were excluded from the analysis to minimize the confounding effects of physiological high-frequency activity and noise. Bipolar montage was used to re-reference the SEEG data, and electrode pairs with participation in several anatomical locations were disqualified from analysis. A 120-s continuous data segment, containing fewer than 4 discernible spikes (i.e., < 2 spikes per minute) and free of significant artifacts, was selected for subsequent analysis [14]. This stringent selection criteria was employed to ensure high signal-to-noise ratio and signal stationarity, as interictal epileptiform discharges can induce transient, pathological network synchronizations that may bias functional connectivity estimates. Previous studies have indicated that this duration is sufficient for robust functional connectivity analysis in SEEG data [15].Two epileptologists divided the anatomical region of each contact into “epileptogenic” or “non-epileptogenic” using traditional clinical interpretation. This technique was carried out at the conclusion of the whole SEEG recording and RF-TC session. When the two epileptologists’ conclusion were not unified, the final conclusion would be determined after discussion by all study group members to avoid possible bias. The epileptogenic zone was defined using the criteria suggested by Luders and colleagues [16]. High frequency oscillations, periodic spikes, sharp activity and any other specific electrographic patterns were employed by epileptologists to assist locate epileptogenic locations. We calculated the imaginary coherence (iCoh) to estimate functional connectivity. iCoh ignores signals with zero-time latency and reduces artifacts and volume conduction effects [17]. iCoh was estimated in the delta (0–4 Hz), theta (4–8 Hz), alpha (8–14 Hz), beta (14–30 Hz), gamma (30–80 Hz) bands by Brainstorm. The coherency analysis generated a contact-by-contact iCoh functional connectivity matrix. We defined **'**within-connectivity' as the mean iCoh of electrode contact pairs located within the same functional zone (either the EZ or NEZ). Conversely, 'between-connectivity' was defined as the mean iCoh between electrode contact pairs within a designated functional zone and all other sampled contacts across the remaining brain regions. The classification of these functional zones was determined by clinical seizure semiology and ictal onset patterns recorded via SEEG, rather than strictly following anatomical brain atlases.

We used clustering coefficient, eigenvector centrality, nodal betweenness centrality and edge betweenness centrality to further investigate functional connectivity between specific anatomical sites represented by electrode contacts Clustering coefficient describes the degree of clustering between the regions in brain [18, 19]. Eigenvector centrality measures a node’s importance while giving consideration to the importance of its neighbors [20, 21]. Betweenness centrality is the percentage of all shortest routes in the network that contain a certain node or link between nodes [22, 23]. These investigations made use of an undirected contact-by-contact imaginary coherence matrix. To perform graph theory analysis, the Brain Connectivity Toolbox [24] and custom Matlab scripts were used. BrainNet Viewer was used to display the findings of our betweenness centrality analysis [25].

### Statistical analyses

Before using parametric testing, the Anderson–Darling test for normal distribution was used. Functional connection measurements were compared using 2-tailed paired t-tests between before and after RF-TC. First, independent of the brain area, we analyzed whether RF-TC affects the various functional connection metrics in all contacts. After that, we investigated whether the impact of RF-TC was localized by conducting the same investigation in the epileptogenic zone and non-epileptogenic zone. To facilitate the statistical analysis, we employed the difference between the functional connectivity "after" and "before" RF-TC, which has a distribution that is close to normal. The differences between various circumstances were further compared using a two-way analysis of variance (ANOVA) with Holm-Sidak correction. Statistical analyses were performed using Prism 8.2 (GraphPad Software, San Diego, California). Unless otherwise stated, a p-value was considered significant at 0.05.

## Results

### Patient and data characteristics

Seventeen patients (8 women, 9 men) were included in our study. The mean age at RF-TC was 26.8 ± 9.4 years, and the mean age at epilepsy onset was 17.7 ± 9.4 years, with a mean epilepsy duration was 9.0 ± 5.0 years prior to RF-TC. Before RF-TC, seizure frequency was monthly in nine patients, weekly in four, and daily in four. By the end of the clinical SEEG, seizure onset was localized in temporal region in 8 patients, whereas seizure onset was multilobar in 3 patients. Anatomical extent of epileptogenic zone was limited to sublobar involvement in 7 patients, whereas 10 patients showed seizure onset involving the entire lobe or multiple lobes. Additional information on clinical features and RF-TC procedures in Table 1.

**Table 1:** Summary of Patient Characteristics regarding the stereo-electroencephalography recordings and the clinical response to radiofrequency thermocoagulation

			Mean ± SD/N(%)
**Demographics**			
	Age, years		26.8 ± 9.4
	Gender, female		8 (47.1%)
**Disease information**			
	First seizure age, years		17.7 ± 9.4
	Epilepsy duration, years		9.0 ± 5.0
	Seizure frequency		
		Daily	4 (23.5%)
		Weekly	4 (23.5%)
		Monthly	9 (52.9%)
	Topography of epilepsy		
		Temporal	8 (47.1%)
		Parietal/occipital	3 (17.7%)
		Frontal	2 (11.8%)
		Insulo-opercular	1 (5.9%)
		Multilobar	3 (17.7%)
	Extent of EZ		
		Regional	7 (41.2%)
		Extended	10 (58.8%)
	Drugs		
		VPA	9 (52.9%)
		LVT	12 (70.6%)
		LTG	3 (17.7%)
		CBZ	6 (35.3%)
		PHT	1 (5.9%)
		OXC	11 (64.7%)
		TPM	6 (35.3%)
**Details of recordings**			
	Electrodes implanted		11.2 ± 2.1
	Recording contacts		143.4 ± 29.5
	Contacts in EZ		7.8 ± 2.6
	Contacts in thermocoagulated zone		31.8 ± 20.1
	Clinically improved patients		10 (58.8%)
	Not clinically improved patients		7 (41.2%)

*EZ *epileptogenic zone, *VPA *valproic acid, *CBZ *carbamazepine, *PHT *phenitoin, *OXC *oxcarbazepine, *LVT *levetiracetam, *LTG *lamotrigine, *TPM *topiramate

### Alteration of the functional connectivity after RF-TC

First, regardless of the region of interest or clinical results, we examined whether RF-TC altered the functional connectivity across all recorded channels. In this case, no significant differences were observed between iCoh value nor within iCoh value in any frequency bands.

**Fig. 1:**
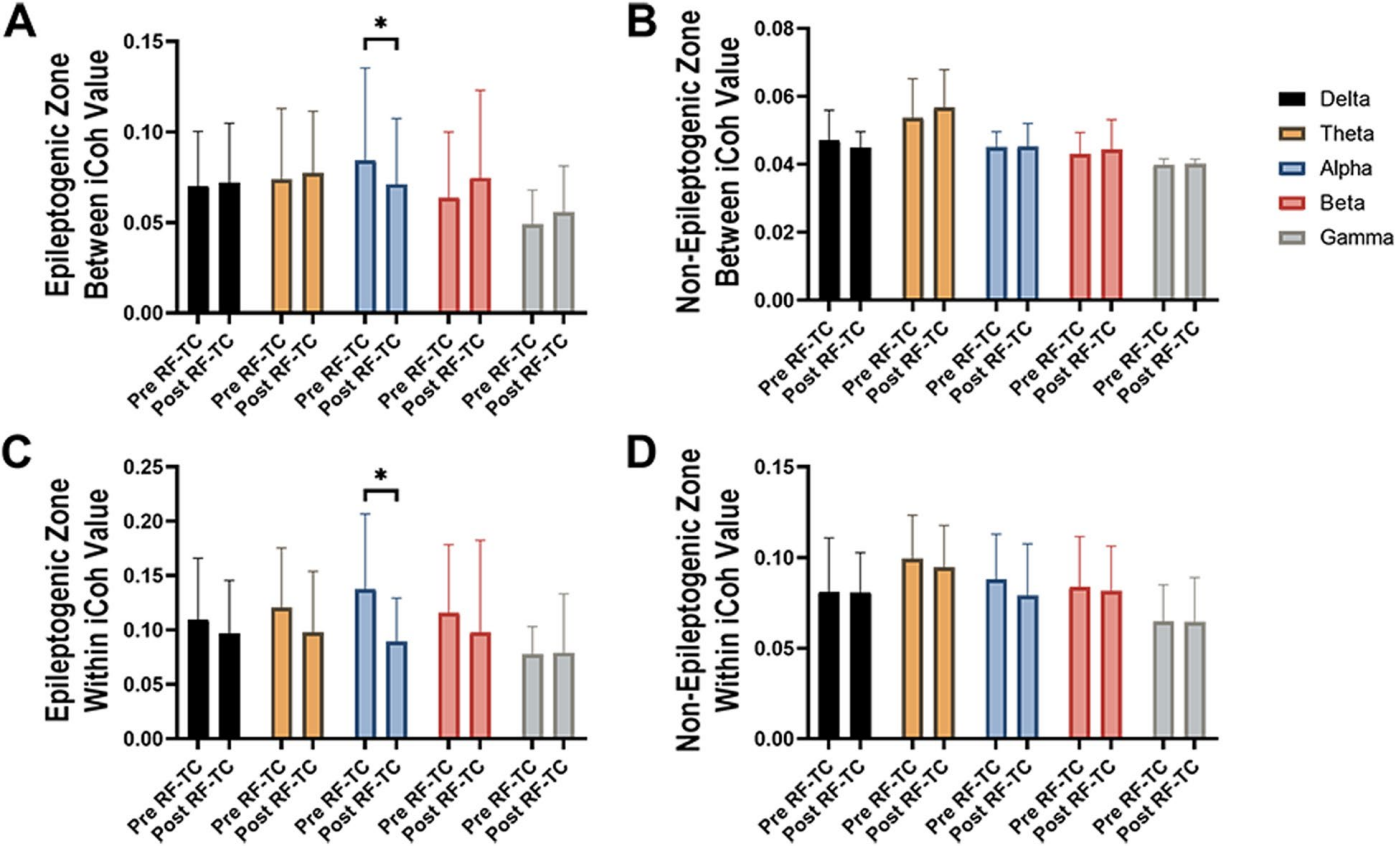
Epileptogenic zone exhibit significant change offunctional connectivityafter radiofrequency thermocoagulation (RF-TC). Across all patients, between connectivity and within connectivity evaluated by imaginary coherrence (iCoh) in epileptogenic zone (A and C) and non-epileptogenic zone (B and D) in different frequency bands. Bars represent the mean ± standard error of the mean between patients, t-test, *p < 0.05. Pre RF-TC, functional connectivity estimated before RF-TC; Post RF-TC, functional connectivity estimated after RF-TC

Next, we analyzed the epileptogenic zone and non-epileptogenic zone separately to determine whether the effect of RF-TC were region-specific (Fig. 1). In the epileptogenic zone, both between connectivity (*p* = 0.0264) and within connectivity (*p* = 0.0116) were significantly reduced in alpha band after RF-TC (Fig. 1A, C). In contrast, no significant modification changes of the functional connectivity were shown in non-epileptogenic zone in any frequency bands (Fig. 1B, D).

### Modification of the network properties after RF-TC

**Fig. 2:**
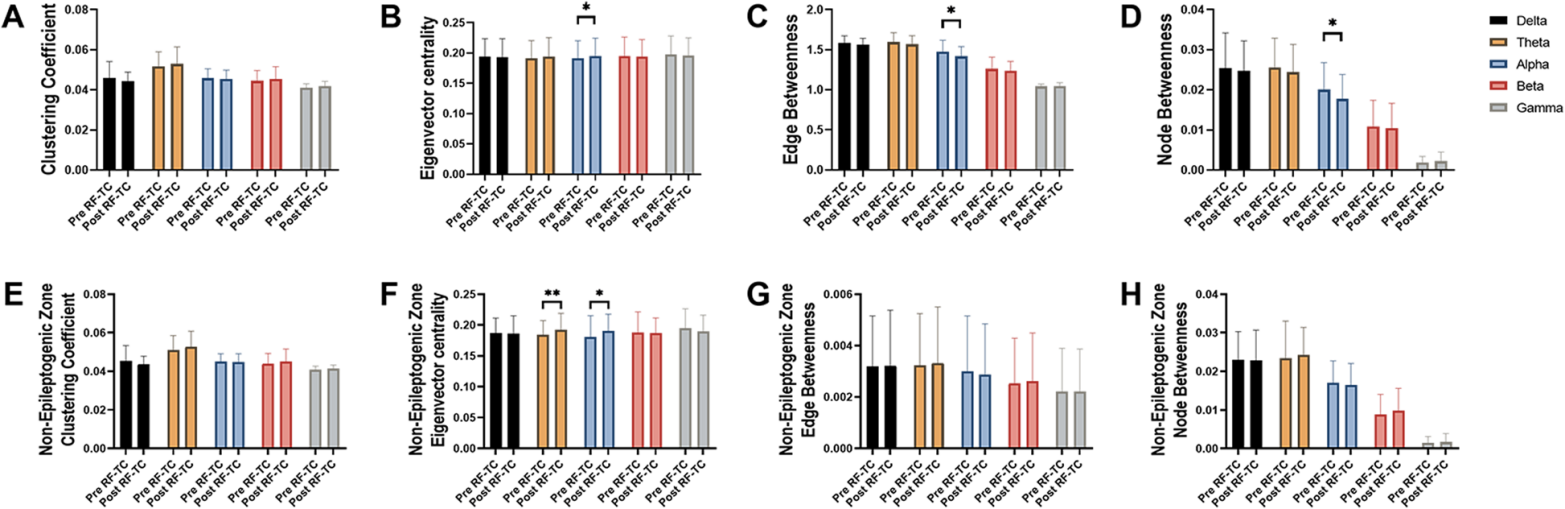
Graph theory analysis of the modification of the functional connectivity after radiofrequency thermocoagulation (RF-TC). A-D: alteration of clustering coefficient, eigenvector centrality, edge betweenness and node betweenness after RF-TC procedure in different frequency bands independent of region of interest; E–F: modification of clustering coefficient, eigenvector centrality, edge betweenness and node betweenness after RF-TC procedure in different frequency bands in non-epileptogenic zone. Bars represent the mean ± standard error of the mean between patients, t-test, *p < 0.05. Pre RF-TC, functional connectivity estimated before RF-TC; Post RF-TC, functional connectivity estimated after RF-TC

**Fig. 3:**
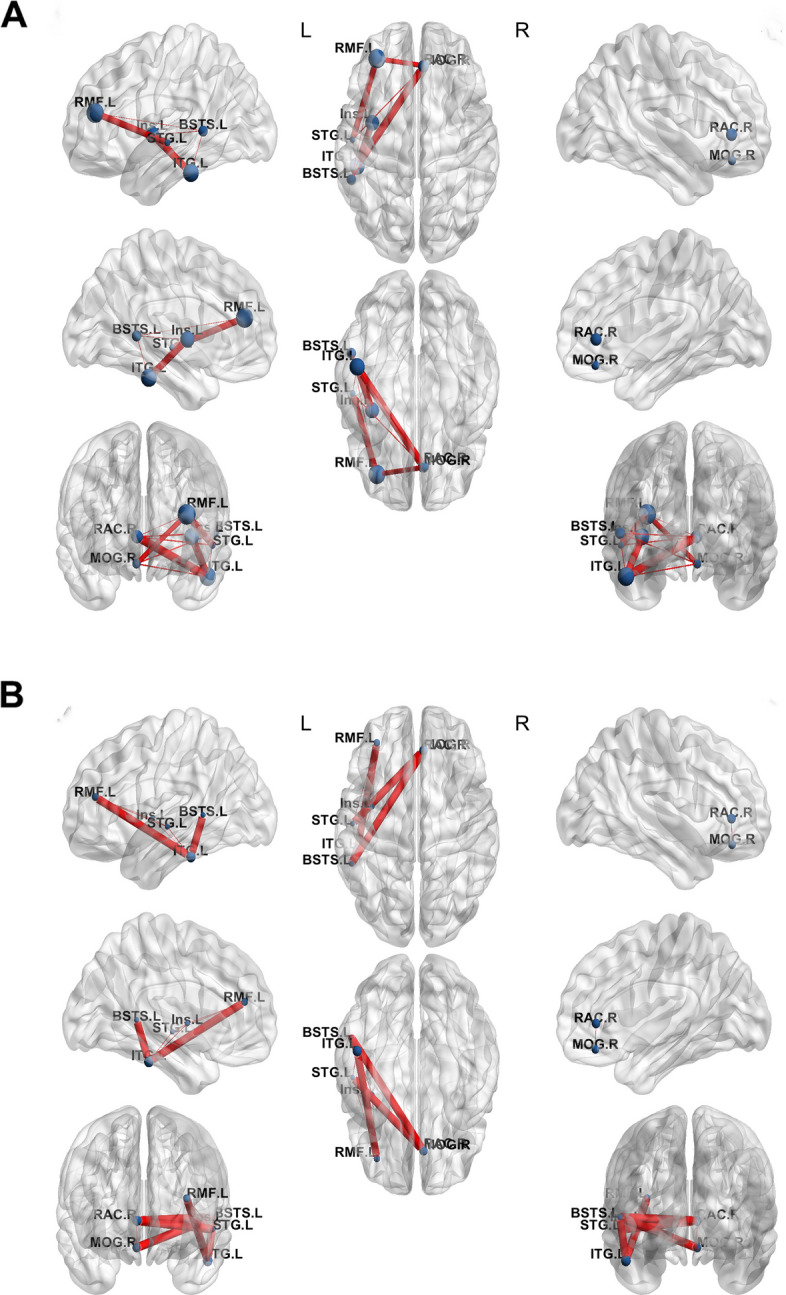
Betweennesscentrality map in an example patient demonstrating alteration of functional connectivity before (A) and after (B) radiofrequency thermocoagulation (RF-TC). 29 years old male patient with bilateral electrode placement. After RF-TC, the overall nodal betweenness centrality (size of sphere) and edge betweenness centrality (thickness of line connecting between 2 spheres) were relatively reducing. Alpha-band imaginary coherence is utilized for all measures. L, left; R, right; Ant, anterior; Post, posterior; STG.L, left superior temporal gyri; ITG.L, left inferior temporal gyri; BSTS.L, left bankssts; RMF.L left rostral middle frontal gyri; Ins.L left insular lobe; MOG.R, right medial orbitofrontal gyri; RAC.R, right rostral anterior cingulate

We used graph theoretical analysis to examine whether RF-TC might possess network properties. As shown in Fig. 2A–D, significant changes were shown in four measurements regardless of region of interest, except for clustering coefficient. In detail, we observed an increase in eigenvector centrality in the alpha band after RF-TC (*p* = 0.0278, Fig. 2B), while a reduction of edge betweenness (*p* = 0.0154, Fig. 2C) and node betweenness (*p* = 0.0122, Fig. 2D) also in the alpha band after RF-TC (Fig. 3).

To determine whether RF-TC–related network changes were localized, we analyzed the epileptogenic and non-epileptogenic zones separately.. We observed no significant changes in any of the four measurements in epileptogenic zones. Surprisingly, in non-epileptogenic zones (Fig. 2F), RF-TC procedure led to a significant increase in eigenvector centrality in theta band (*p* = 0.0055) and the alpha band (*p* = 0.0463).

### Changes in functional connectivity and their relationship to clinical response of RF-TC

**Fig. 4:**
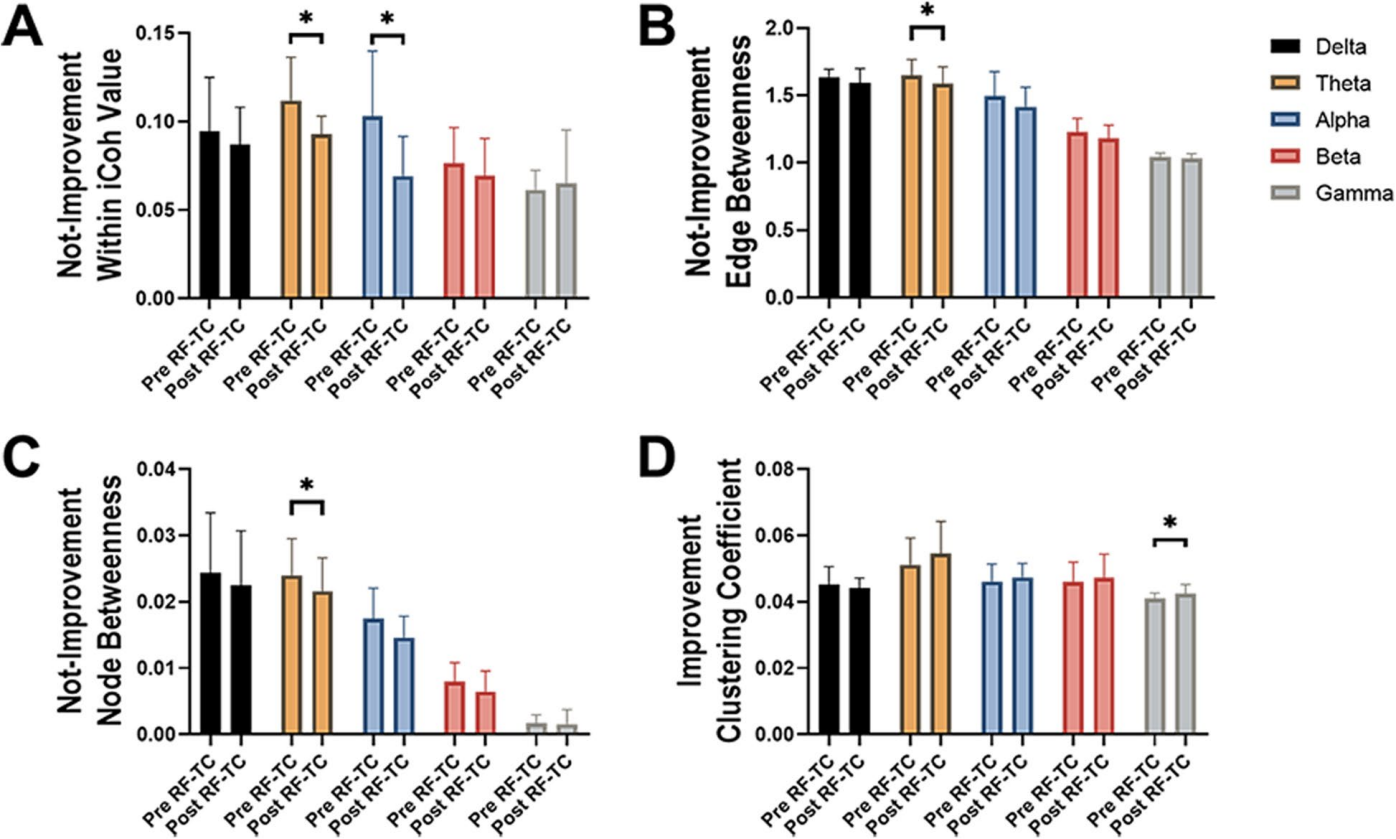
Functional connectivity modification according to clinical response to radiofrequency thermocoagulation (RF-TC). A-C: changes of within connectivity, edge betweenness and node betweenness in patients with no clinical benefit in different frequency bands after RF-TC. D: alteration of clustering coefficient in patients with clinically improved. Bars represent the mean ± standard error of the mean between patients, t-test, *p < 0.05. Pre RF-TC, functional connectivity estimated before RF-TC; Post RF-TC, functional connectivity estimated after RF-TC

In our study, ten patients showed clinical improvement after RF-TC. We therefore tested whether the effect of RF-TC were associated with alteration in functional connectivity. We found significant changes after RF-TC (Fig. 4). Specifically, in non-improvement patients, we observed that within connectivity was decreased significantly in theta (*p* = 0.0341) and alpha (*p* = 0.0439) band after RF-TC (Fig. 4A). Moreover, in same group,edge betweenness (*p* = 0.0418) and node betweenness (*p* = 0.0354) were also significantly decreased in theta band after RF-TC (Fig. 4B, C) i. Among patients with clinical improvement following RF-TC, however, the clustering coefficient increased in the gamma band (*p* = 0.0298, Fig. 4D).

**Fig. 5:**
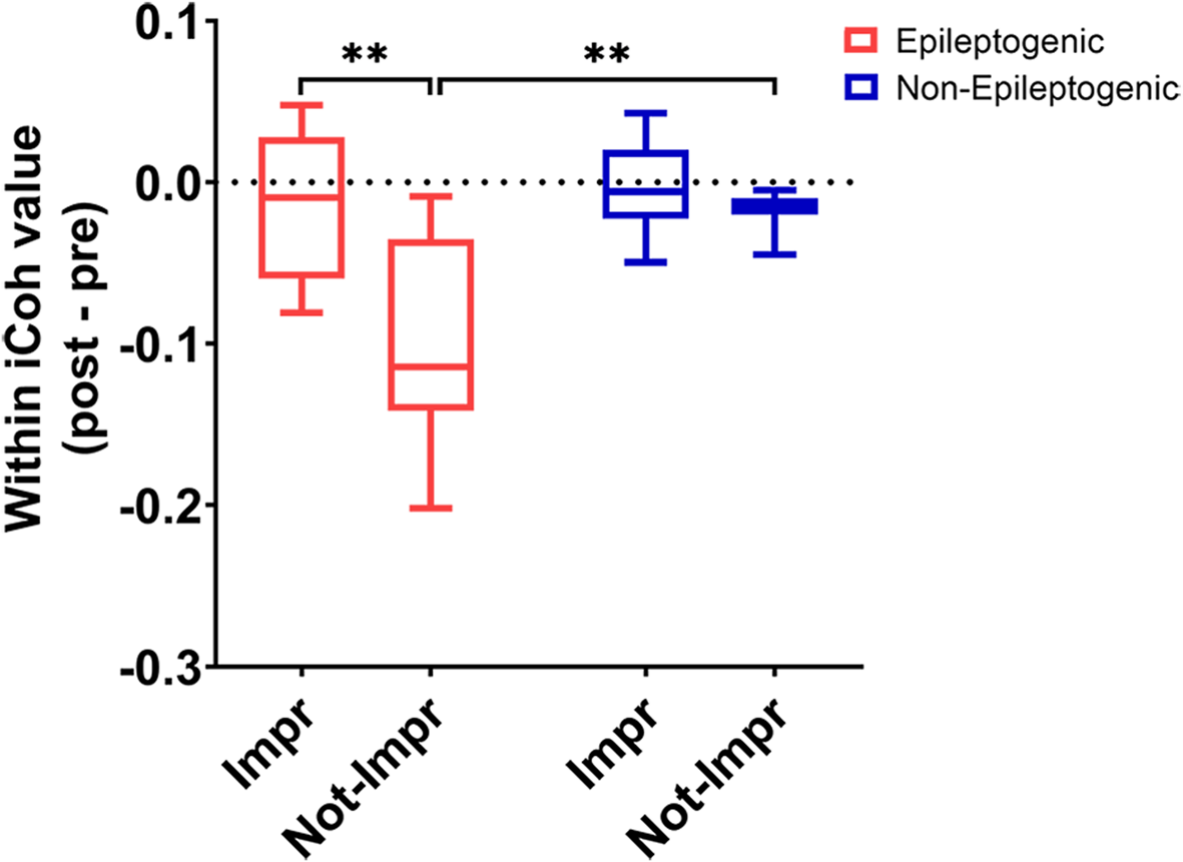
The association between clinical response to radiofrequency thermocoagulation (RF-TC) and within connectivity in the different zones. Comparison between the difference between after and before values in clinically improved versus non-improved patients. Two-way analysis of variance (ANOVA) followed by Holm-Sidak correction, **p < 0.01, bars represent the mean ± standard error of the mean between patients. Impr, clinically improved after RF- TC; Not-impr, not clinically improved after RF- TC

Subsequently, we determined the relationship between clinical response to RF-TC and several functional connectivity markers after thermocoagulation across the different brain regions. We found that only within connectivity in alpha band existed significant change, Fig. 5 illustrates a significant group-level reduction in alpha-band within-connectivity following RF-TC. However, this overarching trend was accompanied by notable inter-subject variability, particularly within the improvement group, where a subset of individuals exhibited a paradoxical increase in connectivity values. Furthermore, the reduction in the epileptogenic zone was smaller in within connectivity in epileptogenic zone in clinically improved patients compared to those who had no clinical improvement (*p* = 0.0021). In contrast, within connectivity showed a greater decrease in epileptogenic zone among patients without clinical improvement (*p* = 0.0063).

## Discussion

In this study, we detected decreased functional connectivity in the epileptogenic zone after RF-TC by using resting-state SEEG data to measure the imaginary coherence in the alpha band. Compared to before RF-TC, all patients demonstrated increased eigenvector centrality and reduced betweenness in alpha band. However, when we tested whether the change was region-specific, we found that the RF-TC procedure only increased the eigenvector centrality in the theta and alpha band. Moreover, we identified that there existed correlation between the modification of functional connectivity and the clinical response to RF-TC. Precisely, within connectivity was considerably reduced in theta and alpha bands following RF-TC in non-improvement group, as were edge betweenness and node betweenness. Conversely, in patients who improved clinically as a result of RF-TC, the clustering coefficient in the gamma band increased significantly. Furthermore, we observed a much larger decrease of within connectivity in the epileptogenic zone in patients who did not improve clinically after RF-TC.

It’s now recognized that epilepsy is caused by complex networks spanning numerous brain regions and varies in size [26]. The majority of investigations showed enhanced functional connectivity in the epileptogenic zone, demonstrating that the presence of epileptogenic cortex may be indicated by enhanced short-term functional connectivity [8, 15]. According to Schindler et al., the decrease in functional connectivity is involved in seizure termination [27] Interestingly, our observation was consistent with data obtained in previous research. We found that both between connectivity and within connectivity decreased in epileptogenic zone after RF-TC. Besides, we also observed a greater reduction of within connectivity in non-epileptogenic zone in not-improved patients after RF-TC. To date, previous research has confirmed that functional connectivity is different in the epileptogenic zone, the propagation zone, and the non-epileptogenic zone. When compared to non-epileptogenic zone, within functional connectivity is higher in epileptogenic zone; moreover, epileptogenic zone and propagation zone have significantly higher functional connectivity than with the non-epileptogenic zone [8]. Accordingly, we hypothesize that the more pronounced reduction of within-connectivity in the non-epileptogenic zone among non-responders reflects a widespread, maladaptive network disruption stemming from an insufficiently comprehensive RF-TC target. When the intervention is too localized to sequester the primary epileptogenic core, persistent pathological activity continues to perturb the global functional architecture. Combined with our finding, although RF-TC is an ideal tool for interrupting epileptic networks of epileptogenic cortex [28, 29], we believe that RF-TC should not be limited to the epileptogenic zone; the functional connectivity between the epileptic and non-epileptic zones should also be broken, which may be a practical strategy for improving the clinical efficacy of RF-TC.

Similar to other studies, our research results also revealed changes in certain frequency bands. The alpha frequency band, which is traditionally associated with maintaining a stable brain state and thalamocortical gating [30], showed the most significant changes. In non-responders, there was a sharp and non-specific decrease in alpha-band connectivity across both epileptogenic and non-epileptic areas, indicating a general collapse of network synchrony. Theta oscillations are often involved in long-range limbic communication and can be modulated by interictal activity [31]. In the non-improvement group, we observed a significant reduction in both theta-band within-connectivity and betweenness centrality (Fig. 4). This marked decrease suggests that an unsuccessful intervention may lead to a detrimental disruption of theta-mediated coordination, potentially reflecting the persistent interference of an un-neutralized epileptogenic core on the rest of the brain. While responders exhibited more stable theta patterns, the drastic reduction in non-responders serves as a marker of maladaptive network reconfiguration. Interestingly, we observed a significant increase in the gamma-band clustering coefficient exclusively in the clinical improvement group (Fig. 4D). Gamma oscillations are typically involved in local information processing and high-level cognitive integration. This 're-clustering' suggests that after RF-TC disrupts the pathological epileptic network, the brain may initiate a restorative process, characterized by enhanced local efficiency and more organized functional segregation. Our findings regarding alpha-band modulation align with recent evidence from Simula et al. (2023), though the direction of connectivity changes in non-responders appears divergent. In contrast to the FC increase reported by Bartolomei’s team, our study observed a significant iCoh decrease in non-responders, which likely characterizes a chaotic loss of phase-locking stability rather than therapeutic stabilization. Collectively, these data reinforce the role of alpha-band dynamics as a robust biomarker for evaluating the immediate efficacy of minimally invasive interventions [32].

This orderly coordination of the brain network at specific frequencies indicates that RF-TC plays a role in a precise neural regulation. This phenomenon prompts us to consider what the differences are between RF-TC and traditional resection surgeries in terms of functional connectivity.Although both aim to eliminate the epileptogenic focus, the paths by which they alter the brain network are significantly different. The traditional resection surgery involves the physical removal of large areas of brain tissue and its associated white matter fiber bundles, which typically leads to extensive and non-frequency-specific disruptions of structural and functional connections (PMID: 39,854,170). While RFTC is more akin to a "network hub disruption" technique, by creating focal lesions at the critical nodes with a size of millimeters (typically 3–8 mm), it induces functional decoupling of the epileptic network rather than large-scale structural reconfiguration.. Furthermore, Gula et al. (2025) utilized cortical-cortical evoked potentials (CCEP) to demonstrate the 'distal network effect' of RFTC, with its influence extending up to 85 mm beyond the ablation site. This indicates that the efficacy of RFTC is not solely due to local tissue inactivation, but rather lies in its re-balancing of the connection strength between distant non-ablated nodes [33]. In combination with the emerging virtual brain twin (VBT) technology in 2025 [34], the current evidence supports that RFTC is a precise neural regulation paradigm based on individualized network dynamic simulation, which is fundamentally different from the passive network reconfiguration after resection surgery.

Recent evidence suggests that graph measures play an important role in identifying critical epileptogenic networks. Around the time of seizure onset, the eigenvector centrality calculated from electrocorticographic recordings is a marker of electrodes isolated from the epileptogenic network; however, these isolated electrodes may be able to determine the seizure onset zone with greater specificity and sensitivity [35]. Another group used random-effect models to perform a meta-analysis and discovered that functional networks were similar across patients and controls except for the beta band clustering coefficient [36]. Wilke et al. reported that resection of brain areas with the highest betweenness centrality resulted in a better outcome [18]. More recently, literature has emerged that offers contradictory findings about betweenness centrality, it suggested that recurrent seizures was associated with significant betweenness node removal in certain networks [9]. Additionally, previous study has shown that epileptogenic areas have higher edge betweenness centrality than non-epileptogenic areas [15]. In our study, we found that eigenvector centrality, betweenness centrality and clustering coefficient have significant modification in specific frequency bands after RF-TC. Overall, we and other groups’ finding suggested that a complicated network approach can provide novel ideas for producing quantitative measures that could be utilized as biomarkers or indicators to aid in the early identification of the epileptogenic network and evaluating the effect and efficacy of treatments.

There are some limitations in this research should be considered. Although the results of our functional connectivity analysis were generally similar among the seventeen individuals evaluated, this is a preliminary study with a limited number of patients. Additionally, other vital parts of the epilepsy network, such as the propagation zone and irritative zone, which are not a part of the epileptogenic zone, were not separated in our study, future research may benefit from addressing them. Furthermore, our study concentrated on the short-term effects of RF-TC, since we assessed at the procedure's immediate results. It is important to note that thermocoagulation also have long-term effects. Future research including a bigger cohort with long-term follow-up should examine the relationship between precise functional connection patterns and clinical outcome after RF-TC, in order to evaluate whether network analysis may eventually be utilized to predict clinical response.

## Conclusion

The research on network changes after radiofrequency thermocoagulation (RF-TC) is still an evolving field. Our research results indicate that significant changes occurred in functional connectivity and network characteristics in specific frequency bands and brain regions after RF-TC. Notably, the reduction in the internal connectivity within the alpha frequency band of the epileptic lesion not only provides early electrophysiological feedback for RF-TC, but also serves as a potential indicator for evaluating clinical response and prognosis. In addition, a proof-of-concept finding demonstrated that functional connectivity and graph theory analysis using short resting-state recording epochs may provide helpful prediction data following RF-TC, even if there isn't any ictal or interictal epileptiform activity. While inter-individual heterogeneity suggests these metrics should be interpreted with caution for standalone prediction, the biological consistency observed across independent cohorts reinforces their potential for evaluating the modulatory effects of RF-TC on epileptic networks.

## Supplementary Information

Supplementary Material 1. Supplementary Figure S1: An example ofstereo-electroencephalographyrecording before and after radiofrequency thermocoagulation. The SEEG trace beforeand afterRF-TC showed the obviously reduction of the interictal activity and of tis amplitude. Calibration is in the lower left corner.

## Data Availability

The datasets generated and/or analysed during the current study are not publicly available due to restrictions on data sharing but are available from the corresponding author on reasonable request.

## Abbreviations

SEEG: Stereo-electroencephalography
RF-TC: Radiofrequency thermocoagulation
iCoh: Imaginary coherence
EZ: Epileptogenic zone

## References

[1] Catenoix H, Bourdillon P, Guénot M, et al. The combination of stereo-EEG and radiofrequency ablation. Epilepsy Res. 2018;142:117–20. 29336937 10.1016/j.eplepsyres.2018.01.012

[2] Dimova P, de Palma L, Job-Chapron A-S, et al. Radiofrequency thermocoagulation of the seizure-onset zone during stereoelectroencephalography. Epilepsia. 2017;58:381–92. 28150296 10.1111/epi.13663

[3] Guénot M, Isnard J, Ryvlin P, et al. SEEG-guided RF thermocoagulation of epileptic foci: feasibility, safety, and preliminary results. Epilepsia. 2004;45:1368–74. 15509237 10.1111/j.0013-9580.2004.17704.x

[4] Cossu M, Cardinale F, Casaceli G, et al. Stereo‐<scp>EEG</scp>–guided radiofrequency thermocoagulations. Epilepsia. 2017;1:66–72. 10.1111/epi.13687 28386919

[5] Liscak R, Malikova H, Kalina M, et al. Stereotactic radiofrequency amygdalohippocampectomy in the treatment of mesial temporal lobe epilepsy. Acta Neurochir. 2010;152:1291–8. 20361215 10.1007/s00701-010-0637-2

[6] Contento M, Pizzo F, López-Madrona VJ, et al. Changes in epileptogenicity biomarkers after stereotactic thermocoagulation. Epilepsia. 2021;62:2048–59. 34272883 10.1111/epi.16989

[7] Geller AS, Friedman D, Fang M, et al. Running-down phenomenon captured with chronic electrocorticography. Epilepsia Open. 2018;3:528–34. 30525122 10.1002/epi4.12265 PMC6276771

[8] Lagarde S, Roehri N, Lambert I, et al. Interictal stereotactic-EEG functional connectivity in refractory focal epilepsies. Brain. 2018;141:2966–80. 30107499 10.1093/brain/awy214

[9] Grobelny BT, London D, Hill TC, et al. Betweenness centrality of intracranial electroencephalography networks and surgical epilepsy outcome. Clin Neurophysiol. 2018;129:1804–12. 29981955 10.1016/j.clinph.2018.02.135

[10] Motoi H, Miyakoshi M, Abel TJ, et al. Phase-amplitude coupling between interictal high-frequency activity and slow waves in epilepsy surgery. Epilepsia. 2018;59:1954–65. 30146766 10.1111/epi.14544 PMC6204289

[11] Kahane P, Landré E, Minotti L, et al. The bancaud and Talairach view on the epileptogenic zone: a working hypothesis. Epileptic Disord. 2006;8(Suppl 2):S16-26. 17012069

[12] Tadel F, Baillet S, Mosher JC, et al. Brainstorm: a user-friendly application for MEG/EEG analysis. Comput Intell Neurosci. 2011. 10.1155/2011/879716. 21584256 10.1155/2011/879716 PMC3090754

[13] Cossu M, Fuschillo D, Casaceli G, et al. Stereoelectroencephalography-guided radiofrequency thermocoagulation in the epileptogenic zone: a retrospective study on 89 cases. J Neurosurg. 2015;123:1358–67. 26090841 10.3171/2014.12.JNS141968

[14] Wu JY, Goyal M, Peters JM, et al. Scalp EEG spikes predict impending epilepsy in TSC infants: a longitudinal observational study. Epilepsia. 2019;60:2428–36. 31691264 10.1111/epi.16379 PMC6910957

[15] Goodale SE, González HFJ, Johnson GW, et al. Resting-state SEEG may help localize epileptogenic brain regions. Neurosurgery. 2020;86:792–801. 31814011 10.1093/neuros/nyz351 PMC7225010

[16] Lüders H, Acharya J, Baumgartner C, et al. Semiological seizure classification. Epilepsia. 1998;39:1006–13. 9738682 10.1111/j.1528-1157.1998.tb01452.x

[17] Rolston JD, Chang EF. Critical language areas show increased functional connectivity in human cortex. Cereb Cortex. 2018;28:4161–8. 29045564 10.1093/cercor/bhx271 PMC6215463

[18] Wilke C, Worrell G, He B. Graph analysis of epileptogenic networks in human partial epilepsy. Epilepsia. 2011;52:84–93. 21126244 10.1111/j.1528-1167.2010.02785.x PMC3200119

[19] Hill AT, Hadas I, Zomorrodi R, et al. Modulation of functional network properties in major depressive disorder following electroconvulsive therapy (ECT): A resting-state EEG analysis. Sci Rep. 2020;10:17057. 10.1038/s41598-020-74103-y PMC7555809 33051528

[20] Panzica F, Varotto G, Rotondi F, et al. Identification of the epileptogenic zone from stereo-EEG signals: a connectivity-graph theory approach. Front Neurol. 2013. 10.3389/fneur.2013.00175. 24223569 10.3389/fneur.2013.00175 PMC3818576

[21] Vaughan DN, Rayner G, Tailby C, et al. MRI-negative temporal lobe epilepsy: a network disorder of neocortical connectivity. Neurology. 2016;87:1934–42. 27694267 10.1212/WNL.0000000000003289

[22] Arienzo D, Leow A, Brown JA, et al. Abnormal brain network organization in body dysmorphic disorder. Neuropsychopharmacology. 2013;38:1130–9. 23322186 10.1038/npp.2013.18 PMC3629399

[23] D B, Fss L, Pc R, et al. Evoked directional network characteristics of epileptogenic tissue derived from single pulse electrical stimulation. Hum Brain Mapp. 2018;39:4611–22. 30030947 10.1002/hbm.24309 PMC6220882

[24] Rubinov M, Sporns O. Complex network measures of brain connectivity: uses and interpretations. Neuroimage. 2010;52:1059–69. 19819337 10.1016/j.neuroimage.2009.10.003

[25] Xia M, Wang J, He Y. BrainNet viewer: a network visualization tool for human brain connectomics. PLoS One. 2013;8:e68910. 23861951 10.1371/journal.pone.0068910 PMC3701683

[26] Bartolomei F, Lagarde S, Wendling F, et al. Defining epileptogenic networks: contribution of SEEG and signal analysis. Epilepsia. 2017;58:1131–47. 28543030 10.1111/epi.13791

[27] Schindler KA, Bialonski S, Horstmann MT, et al. Evolving functional network properties and synchronizability during human epileptic seizures. Chaos. 2008;18:033119. 10.1063/1.2966112 19045457

[28] Mirandola L, Mai RF, Francione S, et al. Stereo-EEG: diagnostic and therapeutic tool for periventricular nodular heterotopia epilepsies. Epilepsia. 2017;58:1962–71. 28880999 10.1111/epi.13895

[29] Bourdillon P, Isnard J, Catenoix H, et al. Stereo electroencephalography-guided radiofrequency thermocoagulation (SEEG-guided RF-TC) in drug-resistant focal epilepsy: Results from a 10-year experience. Epilepsia. 2017;58:85–93. 27859033 10.1111/epi.13616

[30] Halgren M, Ulbert I, Bastuji H, et al. The generation and propagation of the human alpha rhythm. Proc Natl Acad Sci U S A. 2019;116:23772–82. 31685634 10.1073/pnas.1913092116 PMC6876194

[31] Cavanagh JF, Frank MJ. Frontal theta as a mechanism for cognitive control. Trends Cogn Sci. 2014;18:414–21. 24835663 10.1016/j.tics.2014.04.012 PMC4112145

[32] Simula S, Garnier E, Contento M, et al. Changes in local and network brain activity after stereotactic thermocoagulation in patients with drug-resistant epilepsy. Epilepsia. 2023;64:1582–93. 37032394 10.1111/epi.17613

[33] Gula J, Slegers RJ, Van Hoof RHM, et al. The impact of radiofrequency thermocoagulation on brain connectivity in drug-resistant epilepsy: insights from stereo-electroencephalography and cortico-cortical evoked potentials. Epilepsia. 2025;66:1260–73. 39831797 10.1111/epi.18270 PMC11997927

[34] Wang HE, Dollomaja B, Triebkorn P, et al. Virtual brain twins for stimulation in epilepsy. Nat Comput Sci. 2025;5:754–68. 40764764 10.1038/s43588-025-00841-6 PMC12457187

[35] Burns SP, Santaniello S, Yaffe RB, et al. Network dynamics of the brain and influence of the epileptic seizure onset zone. Proc Natl Acad Sci USA. 2014;111:E5321-30. 25404339 10.1073/pnas.1401752111 PMC4267355

[36] Slinger G, Otte WM, Braun KPJ, et al. An updated systematic review and meta-analysis of brain network organization in focal epilepsy: looking back and forth. Neurosci Biobehav Rev. 2022;132:211–23. 34813826 10.1016/j.neubiorev.2021.11.028

