# Supplementary figures and images for: Alteration of functional connectivity and network properties after stereo-electroencephalography guided radiofrequency thermocoagulation
Source: Chin Neurosurg J. 2026 Mar 12;12:9. doi: 10.1186/s41016-026-00428-8 (PMC12980985; doi:10.1186/s41016-026-00428-8)

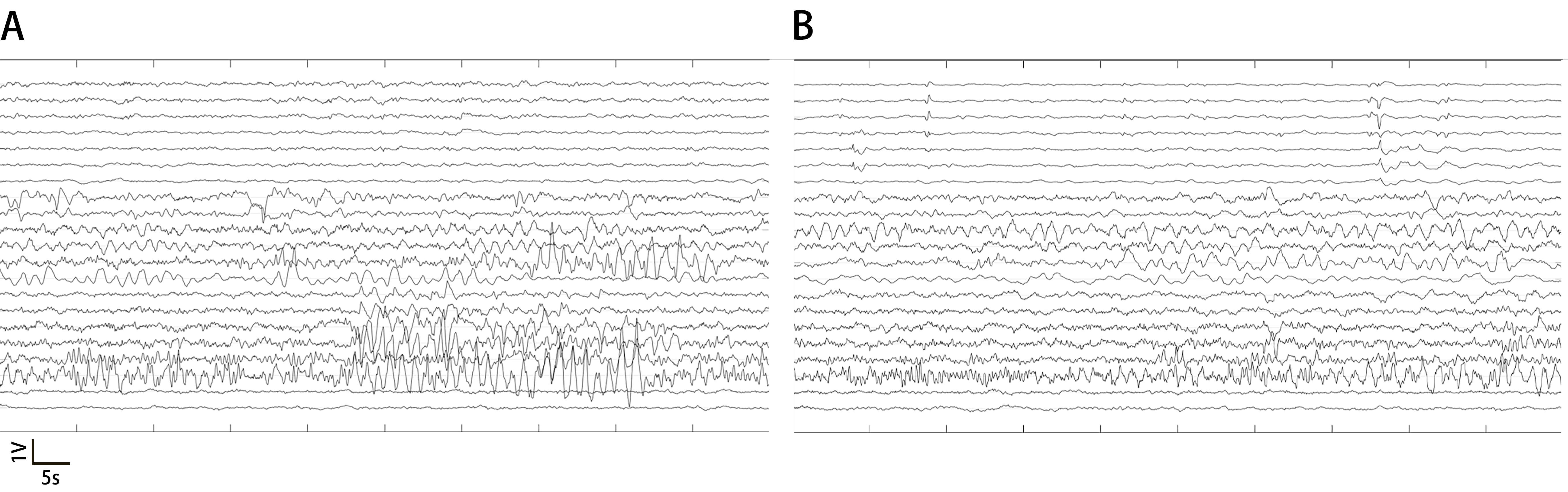

Supplement: Supplementary file 1 — Supplementary Material 1. Supplementary Figure S1: An example ofstereo-electroencephalographyrecording before and after radiofrequency thermocoagulation. The SEEG trace beforeand afterRF-TC showed the obviously reduction of the interictal activity and of tis amplitude. Calibration is in the lower left corner. [file 41016_2026_428_MOESM1_ESM.tif]
